# Boric Acid Exhibits Anticancer Properties in Human Endometrial Cancer Ishikawa Cells

**DOI:** 10.7759/cureus.44277

**Published:** 2023-08-28

**Authors:** Ayşe Çakır Gündoğdu, Neziha Senem Arı, Asiye Höbel, Gülnihal Şenol, Ömer Eldiven, Fatih Kar

**Affiliations:** 1 Histology and Embryology, Kütahya Health Sciences University, Kütahya, TUR; 2 Medical Biochemistry, Kutahya University of Health Sciences, School of Medicine, Kütahya, TUR

**Keywords:** oxidative stress, inflammation, endometrial cancer, boric acid, apoptosis

## Abstract

Objective

This study aims to explore the potential anti-cancer properties of boric acid (BA) in human endometrial cancer Ishikawa cells by assessing its influence on cell viability, apoptosis, oxidative stress, and inflammatory responses.

Methods

The impact of BA at concentrations ranging from 2.5 to 100 mM on cell viability was assessed in Ishikawa cells and normal fibroblast L929 cells (used as the control) through the 3-(4,5-dimethylthiazol-2-yl)-2,5-diphenyltetrazolium bromide (MTT) assay. Spectrophotometric measurements were performed to determine the total oxidant status (TOS) and total antioxidant status (TAS) in BA-treated cells, and the oxidative stress index (OSI) was calculated. The enzyme-linked immunosorbent assay (ELISA) was used to measure the levels of cytochrome c and caspase 3, both of which are constituents of the extrinsic apoptotic pathway. Furthermore, changes in the concentrations of pro-inflammatory cytokines tumor necrosis factor-alpha (TNF-α) and interleukin-1beta (IL-1β) in the cells were analyzed using ELISA and immunofluorescence staining.

Results

The exposure of Ishikawa cells to BA for 24 hours led to a dose-dependent decline in cell viability, with an IC_50_ value of 40 mM. BA dose-dependently increased cytochrome c and caspase 3 levels in cancer cells. In Ishikawa cells, BA treatment led to a significant elevation in OSI. Moreover, the concentrations of TNF-α and IL-1β exhibited a dose-dependent decrease in BA-treated cells. On the other hand, in L929 cells, BA decreased OSI in a dose-dependent manner but did not change TNF-α and IL-1β levels. Concentrations up to 80 mM had no effect on cell viability and apoptosis, but BA at 80 mM concentration decreased viability and increased cytochrome c and caspase 3 levels in L929 cells.

Conclusion

BA inhibited cell viability, triggered apoptosis, induced oxidative stress, and suppressed inflammatory responses in endometrial cancer cells. Notably, at its IC_50_ concentration, BA had no cytotoxic effect on normal fibroblasts. Given its favorable properties, BA may provide a valuable therapeutic option to impede the development and progression of endometrial cancer.

## Introduction

Endometrial cancer, the most common form of gynecological cancer, has experienced an increase in both its incidence and mortality rates over the past few decades [1]. The key contributors to this rise are the utilization of exogenous estrogen and the elevation of endogenous estrogen levels due to factors such as early menarche, nulliparity or less pregnancy, and obesity [2]. While surgical interventions such as hysterectomy have proven effective in treating early-stage endometrial cancer, patients with advanced-stage disease or recurring cases typically require comprehensive treatment methods, yet their prognosis remains unfavorable. Hence, there is a critical need to explore novel therapeutics that possess low toxicity and enhanced efficacy in fighting the disease to treat endometrial cancer as adjuvants to existing therapies. There have been indications that inflammation is implicated in endometrial cancer, and attention has been drawn to the link between the risk of endometrial cancer and the concentration of pro-inflammatory cytokines in the serum [3]. In addition, there have been reports suggesting that the disruption of redox equilibrium caused by the overproduction of reactive oxygen species (ROS) and the decline in antioxidant defense contribute to the initiation and progression of endometrial cancer [4]. Thus, utilizing compounds that display anti-inflammatory activity or that drive cells to apoptosis by increasing oxidative stress is considered a promising and effective strategy for treating endometrial cancer.

Boron, a trace element, is naturally present in water, rocks, and certain soils. It is also found in plant-based food sources consumed by animals and humans, such as grains, nuts, legumes, fruits, and vegetables [5]. It has been reported that plasma boron concentration is approximately 10-20 µM in humans and boric acid (BA) is the predominant soluble form of boron in plasma [6]. BA possesses a broad spectrum of biological effects, and there is growing interest in understanding its impact on health. Studies have indicated that BA contributes to bone health and embryonic development and is beneficial in regulating the inflammatory response and suppressing oxidative stress in various diseases [7]. The bulk of data from preclinical studies suggests that BA serves a crucial role as a tumor suppressor. The therapeutic effectiveness of BA is achieved through mechanisms that involve inhibiting proliferation and angiogenesis, along with triggering apoptosis. Furthermore, it is believed that the anticancer properties of BA are linked to its ability to induce oxidative stress and regulate inflammatory responses [8]. Although there are studies on different cancer types such as breast, colon, and ovarian cancer, especially prostate cancer, the therapeutic potential of BA application in endometrial cancer is not yet known. Therefore, in the current research, we explored the impact of BA on cell viability and apoptotic cell death in endometrial cancer cells and investigated its ability to induce oxidative stress and modulate inflammatory cytokines to understand its potential benefits better.

## Materials and methods

Cell culture

The human endometrial adenocarcinoma Ishikawa cells (99040201) and mouse fibroblast L929 cells (CCL-1) were obtained from the European Collection of Authenticated Cell Cultures (ECACC) and American Type Culture Collection (ATTC), respectively. Ishikawa cells were maintained in RPMI-1640 and L929 cells were cultured in Dulbecco's Modified Eagle Medium (DMEM). In both media, 10% fetal bovine serum (FBS), 100 U/mL of penicillin, and 100 μm/mL of streptomycin were added as supplements. The cells were incubated in a 37°C incubator with 5% CO2 and a humidified atmosphere, and the media were replaced every two days. To test the anticancer effects of BA, Ishikawa, and L929 cells were treated with different BA concentrations (2.5-100 mM) for an additional 24 or 48 h. Cells applied with 0.1% dimethyl sulfoxide (DMSO) served as controls.

Assessment of cell survival

The survival rates of Ishikawa and L929 cells were determined using MTT assay. 5x103 cells were plated in 100 μL of complete media into each well of the 96-well plates and incubated overnight to promote them to become adherent. Cells were then treated with BA dissolved in DMSO at concentrations of 2.5, 5, 10, 20, 40, 80, and 100 mM for 24 and 48 h, based on studies in breast and colon cancer cells [9,10]. Next, the cells were exposed to a 5 mg/mL MTT solution (Elabscience, #E-CK-A341) in 100 μL of culture media and incubated for 4 h. After discarding the medium, 100 µL of DMSO was introduced into each well. The absorbance values of cells in eight replicate wells at 570 nm were measured using a Multiskan™ FC Microplate Photometer.

Total oxidant status (TOS) and total antioxidant status (TAS) measurements

The TOS and TAS measurements were carried out following the instructions provided by the manufacturer (Rel Assay Diagnostics, Gaziantep, Turkey). The TAS measurement involved assessing the conversion of a colored compound called 3-ethylbenzothiazoline-6-sulfonate (ABTS) to a colorless form through the activities of antioxidants present in the Ishikawa and L929 cells. The reduction process was monitored at 660 nm, and the outcomes were reported in mmol Trolox Equiv./L. The TOS present in the cells was quantified by spectrophotometrically measuring the color density of the molecule formed in the reaction medium during the oxidation of ferrous ions to ferric ions at 530 nm. The findings were reported in terms of μmol H2O2 Equiv./L. Oxidative stress index (OSI) was calculated using the formula OSI (arbitrary unit) = (TOS (μmol H_2_O_2_ Eq/L)/TAS (μmol Trolox Eq/L)) ×100.

Assessment of apoptosis and inflammation by enzyme-linked immunosorbent assay (ELISA)

Cytochrome c, caspase 3, TNF-α, and IL-1β concentrations in cells were assessed using commercially available kits that had specific antibodies pre-coated for the respective proteins (Cloud-Clone Corp., USA). Briefly, after rinsing with cold phosphate buffer (pH = 7), the cells were harvested through centrifugation at 1500xg for 10 min. The cells were resuspended in lysis buffer and homogenized for 1 minute. Subsequently, they were centrifuged at 1500×g for 5 minutes at a temperature of +4°C to eliminate cell debris. The resulting cell lysates were preserved at -20°C until they were analyzed.

Cytochrome c, caspase 3, TNF-α, and IL-1β antigen-antibody reactions were performed as per the manufacturer's instructions. The color changes resulting from the reactions were determined spectrophotometrically at a wavelength of 450 nm. By correlating the optical density values with the standard curve, the concentrations of cytochrome c and caspase 3 in the cell lysates were expressed in nanograms per milliliter (ng/mL).

Evaluation of pro-inflammatory cytokine levels by immunocytochemistry

Ishikawa and L929 cells were seeded on round coverslips placed in the wells of 24-well plates and allowed to incubate overnight. Subsequently, cells were exposed to BA at a concentration of 20, 40, and 80 mM. Following a 24-hour incubation period, cells were washed using PBS and fixed by incubation with 4% paraformaldehyde for 20 min at room temperature (RT). Then, cells were rinsed with PBS and treated with 0.1% Triton X-100 for 15 min to induce permeabilization. Following blocking using 3% BSA, cells were incubated overnight at +4ºC with mouse monoclonal anti-TNF-α (Santa Cruz, sc-52746) or anti-IL-1β (Santa Cruz, sc-52012) primary antibodies. On a subsequent day, the cells were washed with PBS and then exposed to Alexa Fluor 488-conjugated goat anti-mouse IgG (Jackson Immuno Research) for one hour at RT. Following the washing cells were mounted with DAPI-containing media (Sigma Aldrich, #F6057) and viewed under a fluorescent microscope (Calibri 7, Zeiss, Germany). Corrected total cell fluorescence (CTCF) was calculated by measuring integrated fluorescence densities in at least 50 cells using ImageJ software [11].

Statistical analysis

GraphPad Prism 8 was employed to conduct the statistical analysis. The data underwent analysis using either the Kruskal-Wallis test or one-way analysis of variance (ANOVA), followed by Tukey's or Dunn's tests. P-values less than 0.05 were regarded as statistically significant. The results were presented as the mean ± standard deviation (SD).

## Results

BA suppresses the survival of endometrial cancer cells

**Figure 1:**
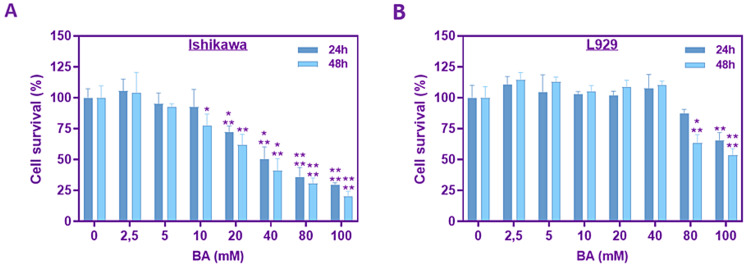
Assessment of cell survival using the MTT assay. The survival rate of the Ishikawa cell line (A) and the L929 cell line (B) after treatment with BA at different concentrations for 24 hours and 48 hours. *p<0.05, **p<0.01, ***p<0.001, and ****p<0.0001.

To explore the potential immediate impacts of BA on cell survival as a measure of cell growth, we initially conducted the MTT assay. As depicted in Figure 1A, exposure to BA for 24 and 48 hours demonstrated a dose-dependent decline in the growth of Ishikawa cells. A significant decrease in cell survival was observed from 20 mM concentration for 24 hours in Ishikawa cells. The half-maximal inhibitory concentration (IC50) of BA was determined to be 40 mM after 24 hours of incubation (p<0.001). Only the highest concentration of BA, which was 100 mM, had a significant effect on reducing the survival of cells after 24 hours when tested on L929 cells (p<0.01). After 48 hours, BA was found to inhibit cell viability starting from a dose of 80 mM (p<0.001), as shown in Figure 1B (p<0.001). Therefore, subsequent experiments focused on BA concentrations of 20, 40, and 80 mM to investigate the dose-dependent impact on cancer cells.

BA induces apoptosis in endometrial cancer cells

**Figure 2:**
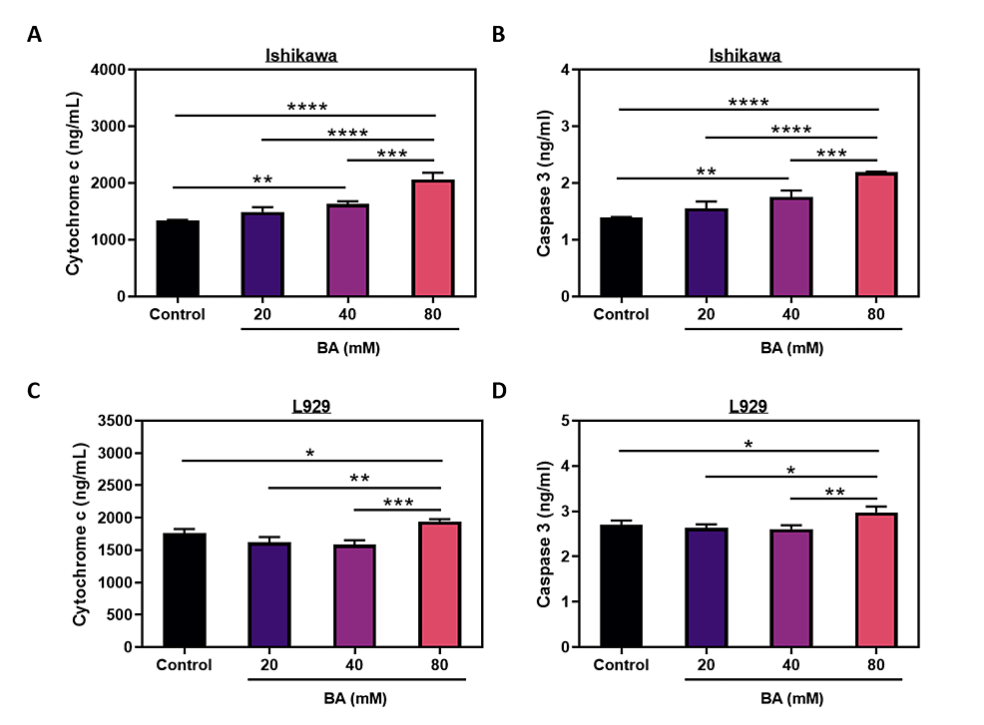
The effect of BA on apoptosis in endometrial cancer. The levels of cytochrome c and caspase 3 in the Ishikawa (A, B) and L929 cells (C, D) under exposure to BA for 24 h. *p<0.05, **p<0.01, ***p<0.001, and ****p<0.0001.

ELISA analyses were conducted to assess the influence of BA on programmed cell death in endometrial cancer. The results revealed that at a concentration of 20 mM, BA did not exhibit a notable effect on cytochrome c levels in Ishikawa cells (Figure 2A, p>0.05). However, at concentrations of 40 and 80 mM, BA significantly elevated cytochrome c levels (p<0.01 and p<0.0001, respectively) (Figure 2A). It was observed that treating L929 cells with BA only at a concentration of 80 mM led to an elevation in both cytochrome c levels (p<0.05) (Figure 2B).

Caspase 3 levels were markedly higher in cells treated with 40 and 80 mM BA for 24 h than in the untreated Ishikawa cells (p<0.01 and p<0.0001, respectively) (Figure 2C). However, similar to cytochrome c, BA at concentrations less than 80 mM had no effect on the caspase 3 levels of L929 cells. At 80 mM concentration, it caused an increase in caspase 3 levels and triggered apoptosis (p<0.05) (Figure 2D).

BA promotes oxidative stress in endometrial cancer cells

**Figure 3:**
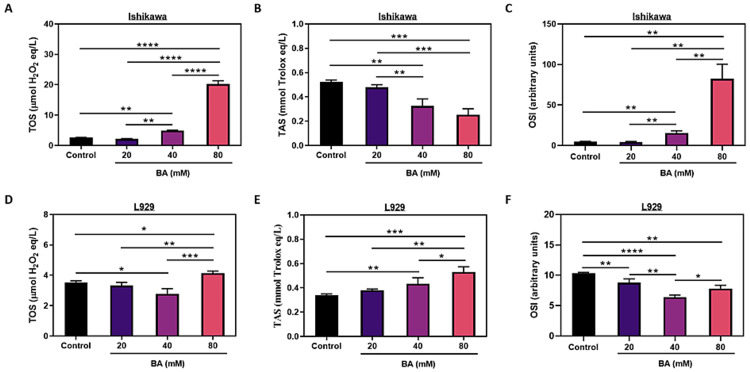
The effect of BA on oxidative stress in endometrial cancer. Analysis of total oxidant status (TOS), total antioxidant status (TAS) and oxidative stress index (OSI) in the Ishikawa (A-C) and L929 cells (D-F) under exposure to BA. *p<0.05, **p<0.01, ***p<0.001, and ****p<0.0001.

To explore the potential involvement of oxidative stress modulation in the anticancer effects of BA, the levels of TOS and TAS were assessed spectrophotometrically. When BA was applied at 40 and 80 mM concentrations to Ishikawa cells, while TOS levels increased (p<0.01 and p<0.0001, respectively) (Figure 3A), TAS levels decreased significantly (p<0.01 and p<0.001, respectively) (Figure 3B). Accordingly, the OSI of Ishikawa cells was significantly elevated at the indicated concentrations (p<0.01). 20 mM BA did not cause any change in oxidative stress (p>0.05) (Figure 3C).

The impact of BA on L929 cells was different from that on Ishikawa cells. While 40 mM BA caused a decline in TOS levels (p<0.05), treatment of 80 mM BA caused a considerable increase in TOS concentrations (p<0.05) (Figure 3D). However, both 40 mM and 80 mM BA treatments led to an elevation in TAS levels (p<0.01 and p<0.001, respectively) (Figure 3E). These alterations were evident in OSI (Figure 3F), showing a significant reduction when compared to the control cells at all three BA concentrations: 20 mM (p<0.01), 40 mM (p<0.001), and 80 mM (p<0.01).

BA reduces TNF-α and IL-1β concentrations in endometrial cancer cells

**Figure 4:**
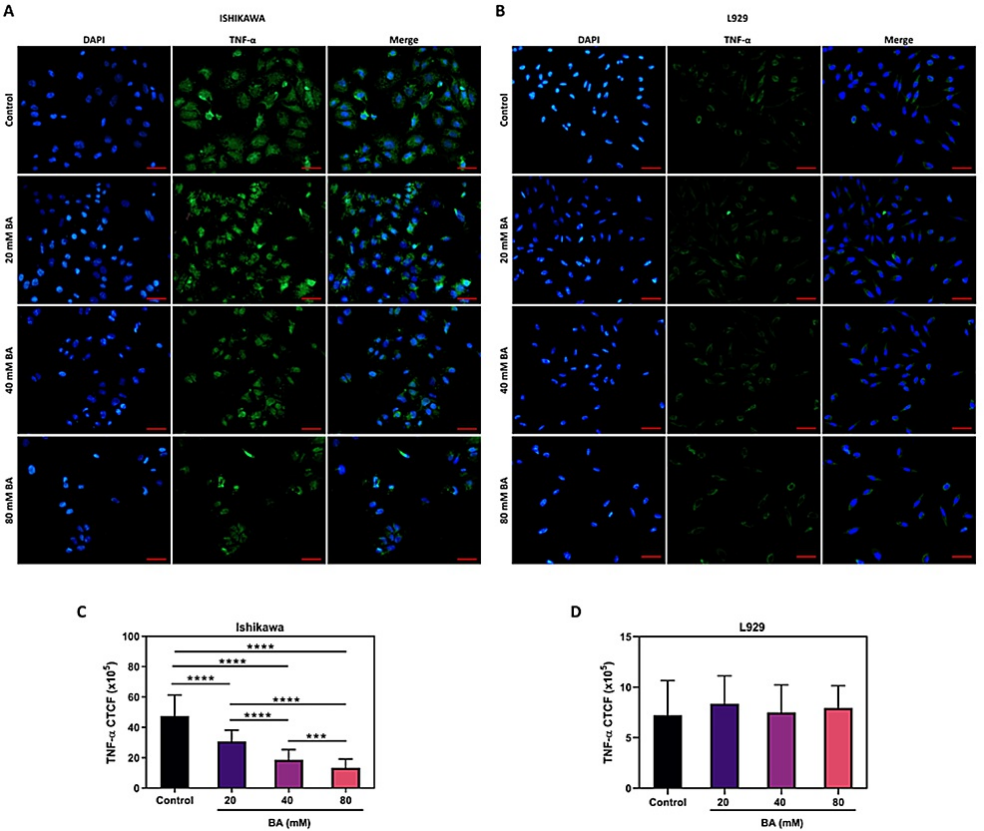
Effect of BA on TNF-α levels in endometrial cancer. Representative images of Ishikawa (A) and L929 (B) cell lines stained with TNF-α antibody (green) following exposure to escalating concentrations of BA for 24 hours. DAPI was utilized for counterstaining nuclei (blue) (scale bar, 100 μm). Bar charts displaying the CTCF of TNF-α in Ishikawa (C) and L929 (D) cells. ***p<0.001, and ****p<0.0001.

**Figure 5:**
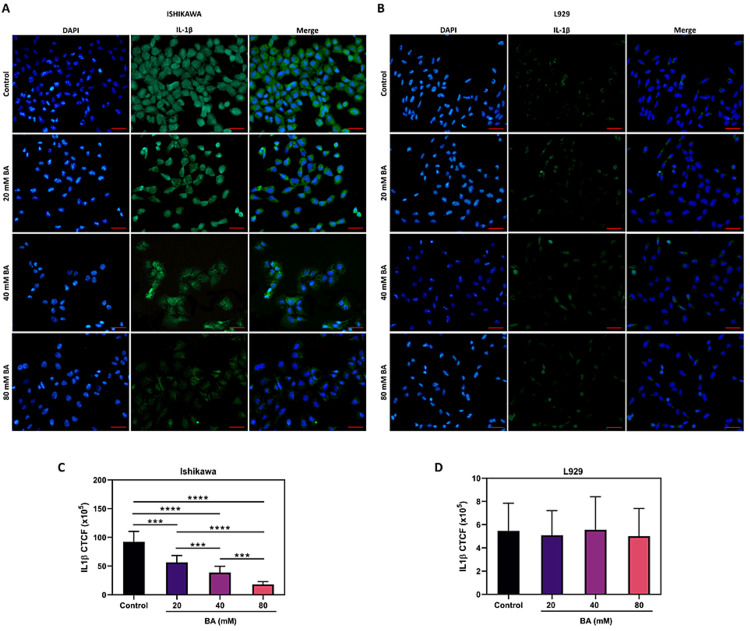
The effect of BA on IL-1β levels in endometrial cancer. Representative images of Ishikawa (A) and L929 (B) cell lines stained with IL-1β antibody (green) following exposure to escalating concentrations of BA for 24 hours. DAPI was utilized for counterstaining nuclei (blue) (scale bar, 100 μm). Bar charts displaying the CTCF of IL-1β in Ishikawa (C) and L929 (D) cells. ***p<0.001, and ****p<0.0001.

In order to examine how BA affects the inflammatory responses in endometrial cancer, we initially assessed the concentrations of the pro-inflammatory cytokines TNF-α and IL-1β using immunofluorescence staining. As illustrated in Figures 4A-4D, BA exhibited a dose-dependent reduction in TNF-α levels specifically in Ishikawa cells (p<0.0001), while none of the BA doses administered to L929 cells resulted in a notable alteration in TNF-α levels (p>0.05). Similarly, as seen in Figures 5A-5D, the intensity of IL-1β staining in Ishikawa cells decreased as the BA dosage increased (p<0.0001), whereas BA did not induce any change in IL-1β intensity in L929 cells (p<0.05).

**Figure 6:**
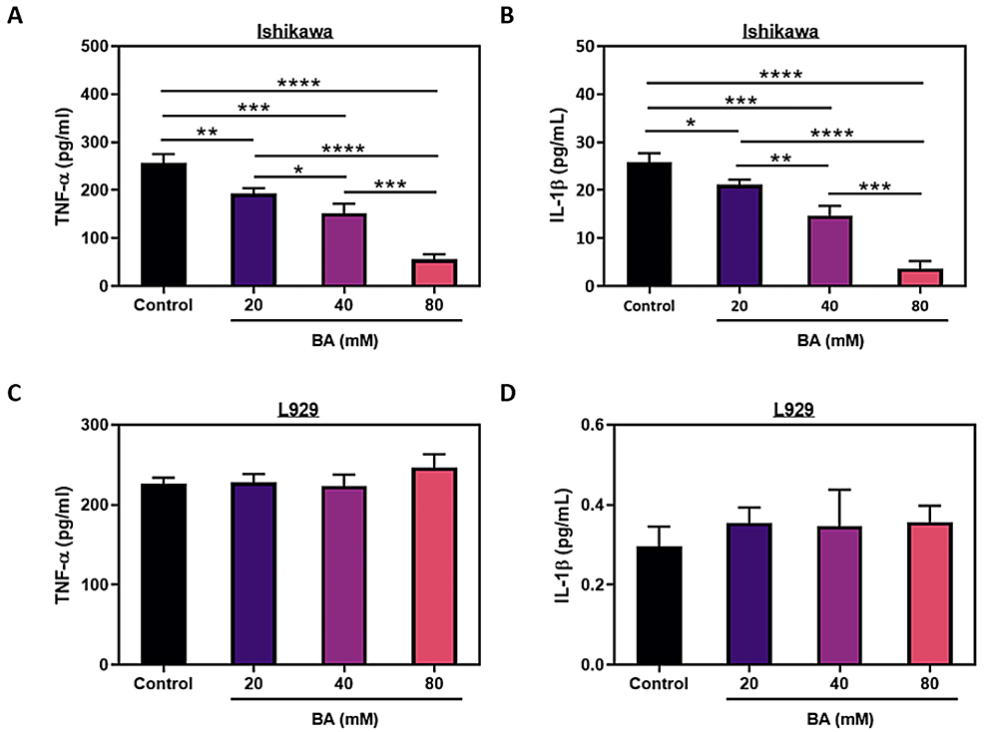
Effect of BA on pro-inflammatory cytokine levels in endometrial cancer. The levels of TNF-α and IL-1β in the Ishikawa (A-B) and L929 cells (C-D) under exposure to increased concentrations of BA. *p<0.05, **p<0.01, ***p<0.001, and ****p<0.0001.

The findings of the ELISA assay, illustrated in Figures 6A-6D, were conducted to validate the immunofluorescence staining data. The findings indicated that in Ishikawa cells, the concentrations of both TNF-α and IL-1β dose-dependently decreased upon the administration of BA. However, no significant variations were detected in the levels of these two cytokines among the various BA concentrations applied.

## Discussion

This study provides the initial evidence highlighting the potential anti-cancer properties of BA in endometrial cancer. We reveal that BA efficiently suppresses the growth of endometrial cancer cells and triggers apoptosis through caspase-dependent mechanisms, with its effects being dose-dependent. Based on our findings, we propose that these anti-cancer effects are likely attributable to the induction of oxidative stress and suppression of the inflammatory responses in cancer cells.

Several reports suggest that inadequate consumption of boron in the diet can lead to an increased risk of cancer among other health issues. Epidemiological investigations have revealed a negative correlation between dietary BA consumption and the risk of developing prostate cancer [12]. Furthermore, a report highlights the hypothesis that Turkey's relatively low rate of cervical cancer cases is attributed to the elevated boron levels found in drinking water [8]. These observations have led to the discovery that BA demonstrates anticancer effects.

One of the primary objectives of cancer therapy is to inhibit the rapid and uncontrolled proliferation of cells. Apart from inhibiting signals related to cell proliferation, the induction of apoptosis plays a crucial role in achieving this objective [13]. In prostate cancer, boron has demonstrated therapeutic potential by reducing the expression of cyclin proteins involved in cell division [14]. Borax, a boron compound, has been shown to induce caspase 3-dependent apoptosis at low concentrations in glioblastoma cells (Hacıoğlu et al., 2023). Additionally, there is evidence showing the effective inhibition of prostate cancer cell growth by BA through its capability to disrupt Ca2+ signaling when present in high concentrations [15]. According to the findings of Scorei et al., exposure to BA led to a suppression of breast cancer cell proliferation. However, it did not lead to an elevation in p53 and bcl-2 protein levels, cytosolic cytochrome c levels, or caspase 3 activity [9]. Conversely, in the study conducted by Kahraman and Göker, it was observed that BA led to an increase in apoptosis and autophagy, inhibited cell viability, and diminished migration in hepatocellular carcinoma cells [16]. In a similar vein, it has been indicated that BA hinders cell proliferation, invasion, migration, and colony formation in ovarian cancer cells, while concurrently promoting apoptosis and inducing oxidative stress [17]. In line with prior research, our study demonstrated for the first time that in endometrial cancer BA led to a significant reduction in cell survival, and this observed effect was contingent on the dosage administered, with an IC50 value of 40 mM. In addition, our findings demonstrated that BA elevated the levels of cytochrome c and caspase 3 in Ishikawa cells by promoting the extrinsic apoptotic pathway. Nevertheless, at a high concentration of 80 mM, BA exhibited cytotoxicity on normal fibroblasts, leading to decreased cell viability and inducing apoptosis. As a result, our findings revealed that BA only at a concentration of 40 mM is safe for normal cells and showed a noteworthy selectivity in targeting cancer cells.

Studies have indicated that oxidative stress is elevated in patients with endometrial carcinoma compared to healthy controls [3]. In the endometrium, both epithelial cells and stromal cells have the capacity to accumulate ROS, and excessive ROS is linked to the development of diseases like endometriosis and polycystic ovary syndrome. In addition, oxidative stress constitutes an important risk factor for malignant transformation because it activates signaling pathways related to cell proliferation [4]. Although ROS promotes tumor growth, excessively high ROS levels become cytotoxic to these cells. Cancer cells increase their antioxidant capacity by activating genes encoding antioxidant system-related enzymes, which enables them to maintain ROS at a level that promotes their growth without undergoing apoptosis. Hence, elevating ROS generation and diminishing antioxidant defenses, i.e., inducing oxidative stress, represent a beneficial approach to promoting apoptosis in cancer cells [18]. Here, we demonstrated that BA induces oxidative stress in a dose-dependent manner in Ishikawa cells by raising their total oxidant levels, while concurrently reducing their total antioxidant capacity. Our findings corroborate the study by Hacıoğlu et al., indicating that BA induces oxidative stress by reducing the levels of antioxidants SOD and CAT [19].

Inflammatory processes are substantial factors in the development and progression of endometrial cancer. Indeed, the fact that estrogen could induce inflammation in the endometrium and the resemblance of cyclic changes in the endometrium to inflammatory processes are among the underlying causes of cancer development in this tissue [20]. The activation of inflammatory reactions prompts rapid cell division in cells and augments the generation of free radicals, which induce damage to DNA [21]. TNF-α and IL-1β, cytokines with a pivotal action in both local and systemic inflammation, are of great importance in cancer development as it is involved in the regulation of cell proliferation and apoptosis. These cytokines also contribute to the progression of cancer by promoting further tumor growth, invasion, and metastasis [22]. TNF-α, generated by both tumor cells and inflammatory cells within the tumor microenvironment, stimulates the expression of growth factors that facilitate cellular proliferation [23]. IL-1β is also recognized as a tumor-promoting cytokine due to its pro-inflammatory properties. IL-1β has been found to contribute to tumor progression by promoting the secretion of matrix metalloproteinases (MMPs) and hepatocyte growth factor (HGF) in lung cancer [24]. Studies have revealed that BA exhibits anti-inflammatory properties in conditions linked to inflammation. For instance, it has been shown that BA inhibits the production of TNF-α induced by lipopolysaccharide in human acute monocytic leukemia cells [25]. It was suggested that BA treatment could regulate inflammation in rats subjected to ovariectomy (OVX) by reducing serum TNF-α levels [26]. Moreover, it has been reported that BA reverses the formaldehyde-induced increase in TNF-α in human lung cancer cells [27]. In this research, we presented novel findings demonstrating that BA, at concentrations of 20 mM and above, had no impact on the quantities of TNF-α and IL-1β in normal fibroblasts. However, in endometrial cancer cells, BA dose-dependently reduced the levels of both cytokines. In a recent study, it has been shown that BA triggers TNF-α-mediated apoptosis in human colon cancer cells [10]. The discrepancy between studies may be due to the difference in cancer types and the activity of other signaling pathways. Besides being anti-apoptotic, TNF-α also has pro-apoptotic properties. Because, the activation of nuclear factor-kappaB (NF-κB), one of the downstream signals of TNF-α, induces cell survival signals, while the sustained activation of another mediator, c-Jun N-terminal protein kinase (JNK), triggers apoptotic cell death [28]. Thus, in endometrial cancer cells, BA probably caused NF-κB inhibition through TNFα inhibition, thereby suppressing cell survival and inducing apoptosis.

## Conclusions

In conclusion, in the current research, we report that BA acts possibly through its oxidative stress-inducing and anti-inflammatory characteristics to induce apoptosis and inhibit cell proliferation in endometrial cancer cells. Our findings support the potential of BA as a therapeutic agent for endometrial cancer. One of the limitations of this study is that the precise mechanism of action of BA at the cellular level in endometrial cancer could not be clarified. The levels of TNF-α in cells under exposure to BA were evaluated however, a comprehensive analysis of the downstream targets of TNF-α was unable to be performed. In addition, total oxidant and antioxidant levels were measured in our study, however, we were unable to identify the specific molecules and enzymes that BA modulates when triggering oxidative stress. Although BA does not exhibit cytotoxicity in normal cells in vitro at a 40 mM concentration, where it demonstrates anticancer properties against cancer cells, its safety, and effectiveness in human cancers need to be evaluated through future in vivo investigations. Additionally, it would be of interest to explore in future studies if BA serves as an adjuvant to conventional chemotherapy.

## References

[1] Brüggmann D, Ouassou K, Klingelhöfer D, Bohlmann MK, Jaque J, Groneberg DA (2020). Endometrial cancer: mapping the global landscape of research. J Transl Med.

[2] Lortet-Tieulent J, Ferlay J, Bray F, Jemal A (2018). International patterns and trends in endometrial cancer incidence, 1978-2013. J Natl Cancer Inst.

[3] Heidari F, Rabizadeh S, Mansournia MA (2019). Inflammatory, oxidative stress and anti-oxidative markers in patients with endometrial carcinoma and diabetes. Cytokine.

[4] Liu Q, Yu M, Zhang T (2022). Construction of oxidative stress-related genes risk model predicts the prognosis of uterine corpus endometrial cancer patients. Cancers (Basel).

[5] Kuru R, Yilmaz S, Tasli PN, Yarat A, Sahin F (2019). Boron content of some foods consumed in Istanbul, Turkey. Biol Trace Elem Res.

[6] Paties Montagner G, Dominici S, Piaggi S, Pompella A, Corti A (2023). Redox mechanisms underlying the cytostatic effects of boric acid on cancer cells-an issue still open. Antioxidants (Basel).

[7] Nielsen FH, Meacham SL (2011). Growing evidence for human health benefits of boron. J Evid Based Complementary Altern Med.

[8] Scorei RI, Popa R Jr (2010). Boron-containing compounds as preventive and chemotherapeutic agents for cancer. Anticancer Agents Med Chem.

[9] Scorei R, Ciubar R, Ciofrangeanu CM, Mitran V, Cimpean A, Iordachescu D (2008). Comparative effects of boric acid and calcium fructoborate on breast cancer cells. Biol Trace Elem Res.

[10] Sevimli M, Bayram D, Özgöçmen M, Armağan I, Semerci Sevimli T (2022). Boric acid suppresses cell proliferation by TNF signaling pathway mediated apoptosis in SW-480 human colon cancer line. J Trace Elem Med Biol.

[11] Gündoğdu AÇ, Özyurt R (2023). Resveratrol downregulates ENaCs through the activation of AMPK in human colon cancer cells. Tissue Cell.

[12] Gonzalez A, Peters U, Lampe JW, White E (2007). Boron intake and prostate cancer risk. Cancer Causes Control.

[13] Kasibhatla S, Tseng B (2003). Why target apoptosis in cancer treatment?. Mol Cancer Ther.

[14] Barranco WT, Eckhert CD (2004). Boric acid inhibits human prostate cancer cell proliferation. Cancer Lett.

[15] Barranco WT, Kim DH, Stella SL Jr, Eckhert CD (2009). Boric acid inhibits stored Ca2+ release in DU-145 prostate cancer cells. Cell Biol Toxicol.

[16] Kahraman E, Göker E (2022). Boric acid exert anti-cancer effect in poorly differentiated hepatocellular carcinoma cells via inhibition of AKT signaling pathway. J Trace Elem Med Biol.

[17] Cabus U, Secme M, Kabukcu C, Cil N, Dodurga Y, Mete G, Fenkci IV (2021). Boric acid as a promising agent in the treatment of ovarian cancer: molecular mechanisms. Gene.

[18] Hayes JD, Dinkova-Kostova AT, Tew KD (2020). Oxidative stress in cancer. Cancer Cell.

[19] Hacioglu C, Kar F, Kacar S, Sahinturk V, Kanbak G (2020). High concentrations of boric acid trigger concentration-dependent oxidative stress, apoptotic pathways and morphological alterations in DU-145 human prostate cancer cell line. Biol Trace Elem Res.

[20] Modugno F, Ness RB, Chen C, Weiss NS (2005). Inflammation and endometrial cancer: a hypothesis. Cancer Epidemiol Biomarkers Prev.

[21] Hussain SP, Hofseth LJ, Harris CC (2003). Radical causes of cancer. Nat Rev Cancer.

[22] Zhao H, Wu L, Yan G, Chen Y, Zhou M, Wu Y, Li Y (2021). Inflammation and tumor progression: signaling pathways and targeted intervention. Signal Transduct Target Ther.

[23] Lau TS, Chan LK, Wong EC (2017). A loop of cancer-stroma-cancer interaction promotes peritoneal metastasis of ovarian cancer via TNFα-TGFα-EGFR. Oncogene.

[24] Yano S, Nokihara H, Yamamoto A (2003). Multifunctional interleukin-1beta promotes metastasis of human lung cancer cells in SCID mice via enhanced expression of adhesion-, invasion- and angiogenesis-related molecules. Cancer Sci.

[25] Cao J, Jiang L, Zhang X, Yao X, Geng C, Xue X, Zhong L (2008). Boric acid inhibits LPS-induced TNF-alpha formation through a thiol-dependent mechanism in THP-1 cells. J Trace Elem Med Biol.

[26] Tekeli H, Ekren Asıcı GS, Bildik A (2022). Anti-inflammatory effect of boric acid on cytokines in ovariectomy-induced rats. Cell Mol Biol (Noisy-le-grand).

[27] Arslan-Acaroz D, Bayşu-Sozbilir N (2020). Ameliorative effect of boric acid against formaldehyde-induced oxidative stress in A549 cell lines. Environ Sci Pollut Res Int.

[28] Wang X, Lin Y (2008). Tumor necrosis factor and cancer, buddies or foes?. Acta Pharmacol Sin.

